# Supplementary material for: Single-cell transcriptomics reveals a mechanosensitive injury signaling pathway in early diabetic nephropathy
Source: Genome Med. 2023 Jan 10;15:2. doi: 10.1186/s13073-022-01145-4 (PMC9830686; doi:10.1186/s13073-022-01145-4)
Supplement: Supplementary file 1 — Additional file 1: Figure S1. Characterization of BTBR ob/ob podocyte-reporter mice. Figure S2. Overview of the single cells from DN and control kidney samples. Figure S3. Distributions of cells across conditions and individual kidney samples. Figure S4. FACS analysis of podocytes (podo), glomerular endothelial cells (gECs) and mesangial cells (mesan) from the isolated mouse glomeruli. Figure 5S. Bulk RNA-seq results for FACS-sorted glomerular cells. Figure S6. Transcriptional regulations in DN mice. Figure S7. snRNA-seq of early human diabetic nephropathy and ex-vivo-perfused pig kidney tissue. Supplementary methods: FACS-sorted glomerular cells. Urine and serum analysis. Histological and ultrastructural analysis. [file 13073_2022_1145_MOESM1_ESM.docx]

**Additional file 1**

**Single-cell transcriptomics reveals a mechanosensitive injury signaling pathway**

**in early diabetic nephropathy**

*Shuya Liu^1, 12,^ *, Yu Zhao^1, 2, 10,12^, Shun Lu^1, 12^, Tianran Zhang^2^, Maja T. Lindenmeyer^1^, Viji Nair^3^, Sydney E. Gies^1^, Guochao Wu^1^, Robert G. Nelson^4^, Jan Czogalla^1^, Hande Aypek^1^, Stephanie Zielinski^5^, Zhouning Liao^1^, Melanie Schaper^1^, Damian Fermin^3^, Clemens D. Cohen^6^, Denis Delic^7,8^, Christian F. Krebs^9,10^, Florian Grahammer^1^, Thorsten Wiech^11^, Matthias Kretzler^3^, Catherine Meyer-Schwesinger^5^, Stefan Bonn ^2,10^, Tobias B. Huber^1, 10,^**

^1^III. Department of Medicine, University Medical Center Hamburg-Eppendorf, Hamburg, Germany

^2^Institute of Medical Systems Biology, University Medical Center Hamburg-Eppendorf, Germany

^3^Department of Internal Medicine, Division of Nephrology, University of Michigan, Ann Arbor, Michigan, USA

^4^Chronic Kidney Disease Section, National Institute of Diabetes and Digestive and Kidney Diseases, National Institutes of Health, Phoenix, AZ, USA

^5^Institute of Cellular and Integrative Physiology, University medical Center Hamburg-Eppendorf, Germany

^6^Nephrological Center, Medical Clinic and Policlinic IV, University of Munich, Munich, Germany

^7^Boehringer Ingelheim Pharma GmbH & Co. KG, Translational Medicine & Clinical Pharmacology, Birkendorferstr. 65, 88397 Biberach, Germany

^8^Fifth Department of Medicine (Nephrology/Endocrinology/Rheumatology), University Medical Centre Mannheim, University of Heidelberg, Germany

^9^Division of Translational Immunology, III. Department of Medicine, University Medical Center Hamburg-Eppendorf, Germany

^10^Hamburg Center for Translational Immunology, University Medical Center Hamburg-Eppendorf, Germany

^11^Institute of Pathology, Nephropathology Section, University Medical Center Hamburg-Eppendorf, Hamburg, Germany.

^12^These authors contributed equally

*Correspondence:

Shuya Liu, PhD

E-mail: [s.liu@uke.de](mailto:s.liu@uke.de)

and

Tobias B. Huber, MD

E-mail: t.huber@uke.de

University Medical Center Hamburg Eppendorf

III. Department of Medicine

Martinistrasse 52

20246 Hamburg

# Supplementary figures


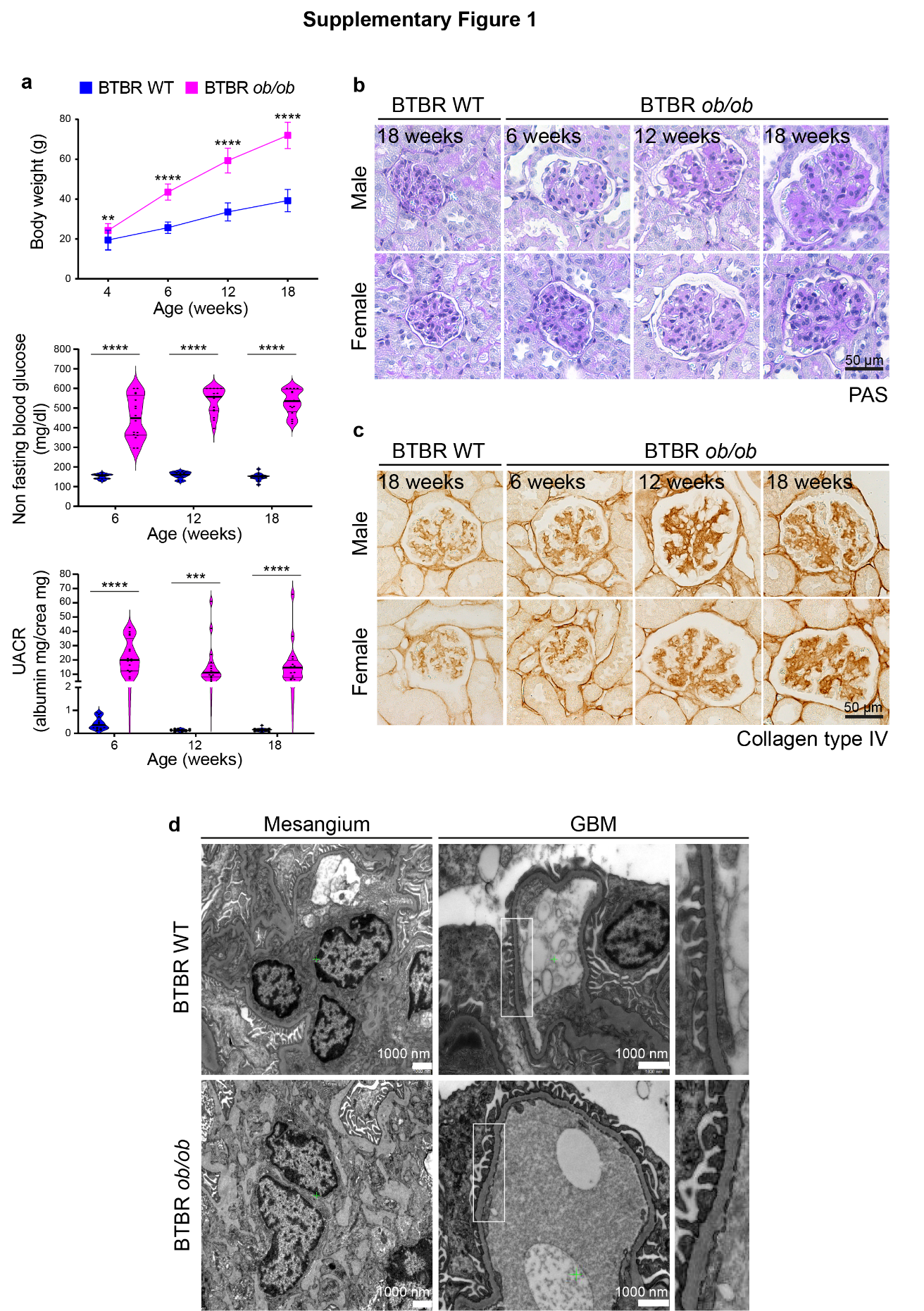


## Fig. S1: Characterization of BTBR ob/ob podocyte-reporter mice

(**a**) Body weights, non fasting blood glucose levels and urine albumin-to-creatinine ratios (UACR) of BTBR *ob/ob* podocyte-reporter mice compared to BTBR WT podocyte-reporter controls (n=16 in each condition; the points and error bars represent the means ± SDs; **p < 0.01, ***p < 0.001, ****p < 0.0001, multiple t tests). (**b**) PAS staining of kidney sections showing obvious glomerular hypertrophy and mesangial expansion in BTBR *ob/ob* podocyte-reporter mice from 12 weeks of age. (**c**) Immunohistochemical staining against collagen IV in kidneys showing increased collagen IV in the glomeruli of BTBR *ob/ob* podocyte-reporter mice from 12 weeks of age. Scale bar: 50 µm (**b, c**). (**d**) Electron microscopy images of glomeruli of 18-week-old BTBR WT and BTBR *ob/ob* podocyte-reporter mice. Mesangial expansion, slight but not obvious thickened glomerular basement membrane (GBM) were observed in BTBR *ob/ob* podocyte-reporter mice.


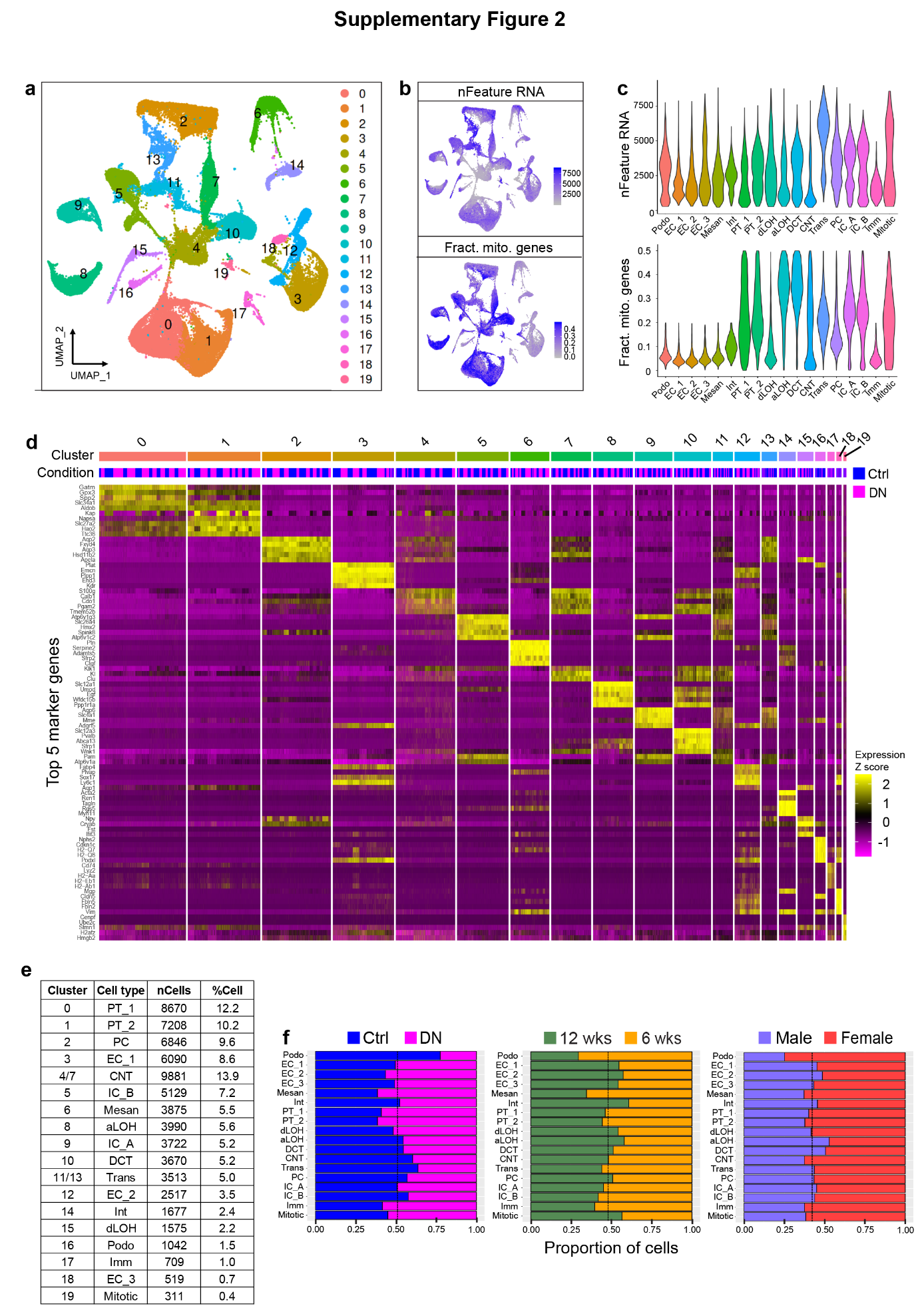


## Fig. S2: Overview of the single cells from DN and control kidney samples

(**a**) UMAP plot for 70,944 cells from all kidney samples (n=16) demonstrating 20 clusters (0-19). The number of genes (nFeature RNA) and the fraction of mitochondrial genes (Fract. Mito. Genes) are displayed in the UMAP plot (**b**) and are profiled in each cell type in violin plots (**c**). (**d**) Heatmap showing the expression levels of top 5 unbiased marker genes in each cluster. (**e**) Annotation of each cluster and the number and percentage of cells in each cell type separated into DN and control groups. (**f**) Proportions of cells from control and DN mice, at 6 weeks and 12 weeks, from male and female mouse kidneys. The dashed lines indicate the average proportion of all cell types.


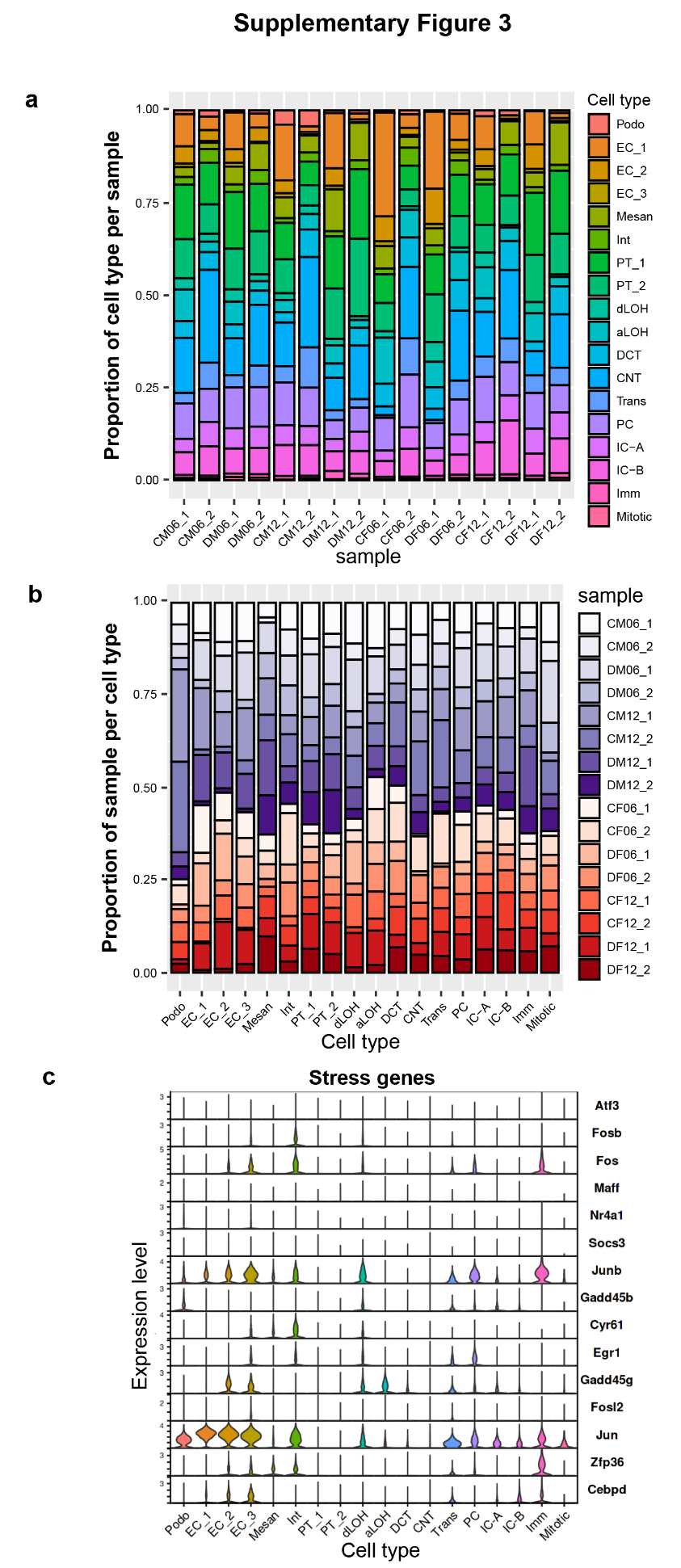


## Fig. S3: Distributions of cells across conditions and individual kidney samples

(**a**) Proportions of cell types for each of the 16 samples. (**b**) Proportions of samples for each of the 18 cell types. CM06: control male at 6 weeks, DM06: DN male at 6 weeks, CM12: control male at 12 weeks, DM12: DN male at 12 weeks, CF06: control female at 6 weeks, DF06: DN female at 6 weeks, CF12: control female at 12 weeks, DF12: DN female at 12 weeks. (**c**) Violin plots showing the expression levels of stressed genes related to single-cell preparation reported by previous studies [1, 2].


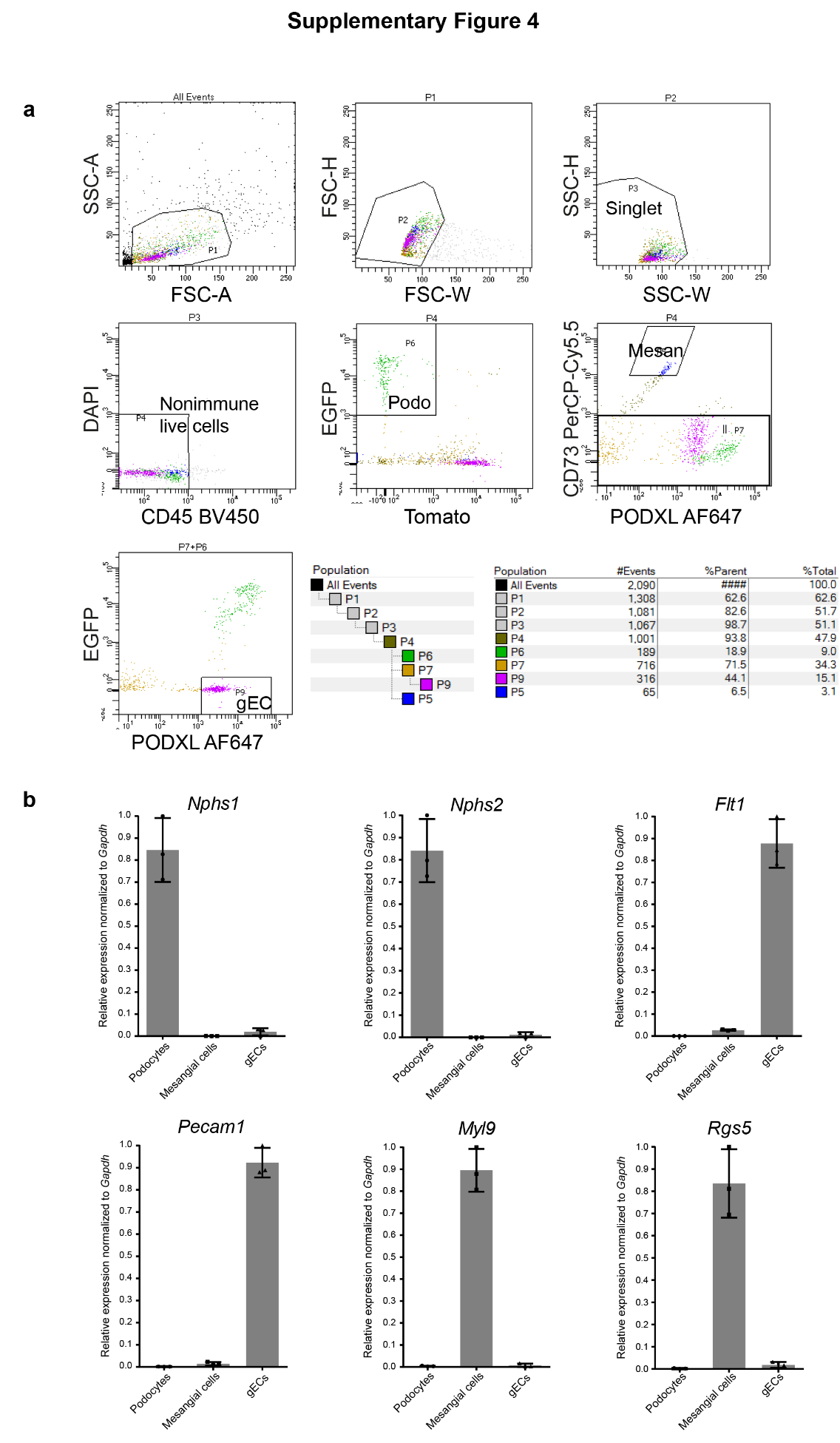


## Fig. S4: FACS analysis of podocytes (podo), glomerular endothelial cells (gECs) and mesangial cells (mesan) from the isolated mouse glomeruli.

(**a**) Representative FACS-sorting dot plots. The cells were stained with antibodies against CD45 coupled to BV450, PODXL coupled to AF647 and CD73 coupled to PerCP-Cy5.5. Nonimmune live single cells (P4) were gated by SSC/FSC and DAPI/CD45. The green dots (P6) were gated from P4 and represent podocytes, which were EGFP positive/Tomato negative/CD73 negative. The blue dots (P5) were gated from P4 and represent mesangial cells, which were CD73 positive/PODXL negative/EGFP negative. The red dots (P9) were gated from P7 (EGFP-negative cells gated from P4) and represent gECs, which were PODXL positive/EGFP negative/CD73 negative. (**b**) Real-time qPCR results of glomerular cell marker genes showing the purity of FACS-sorted cell samples. *Nphs1* and *Nphs2* are podocyte markers. *Flt1* and *Pecam1* are EC markers. *Myl9* and *Rgs5* are mesangial cell markers. n=3 in each condition; the points and error bars represent the means ± SDs.


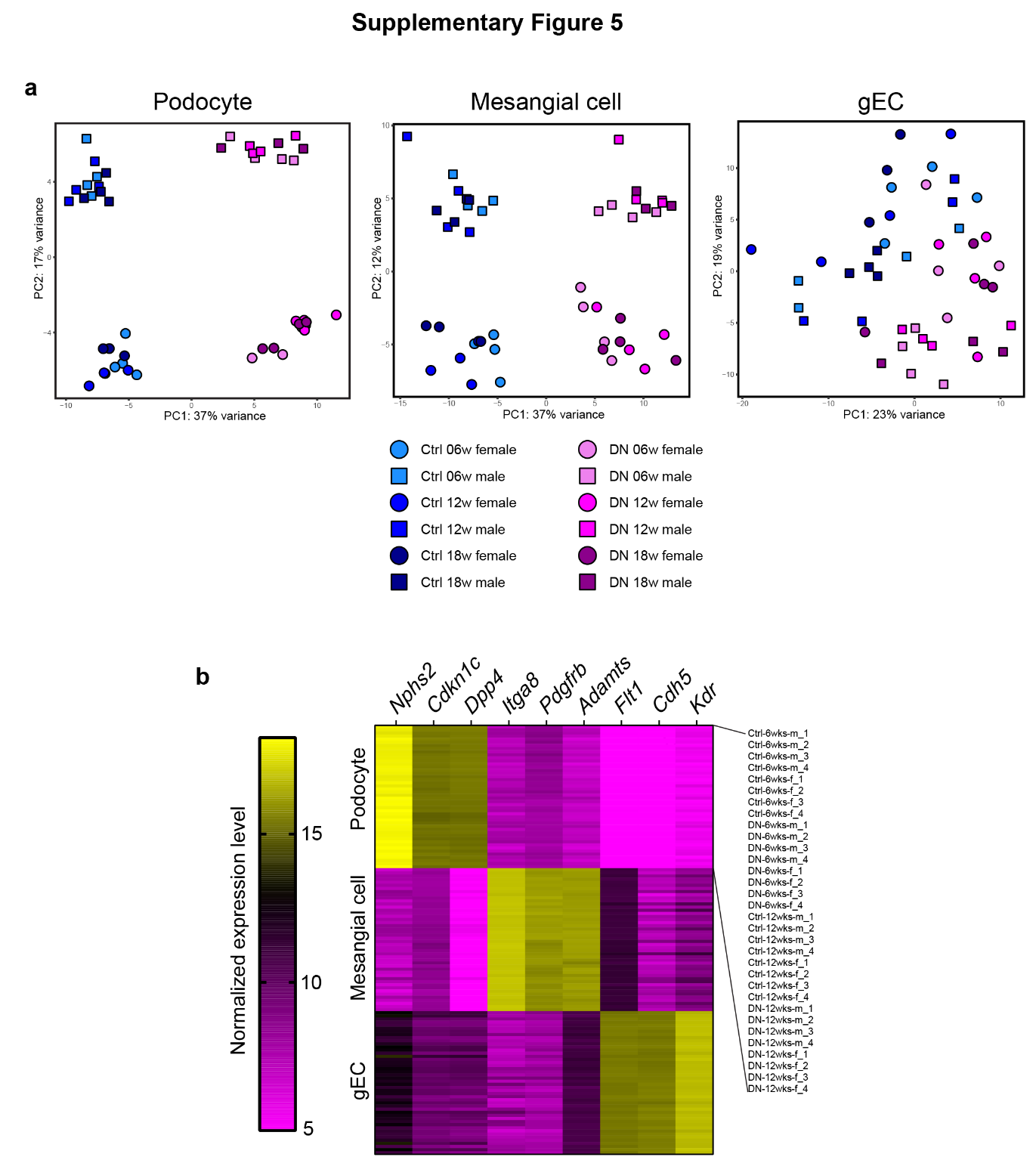


## Fig. 5S: Bulk RNA-seq results for FACS-sorted glomerular cells

(**a**) PCA plots of podocytes, mesangial cells and gECs from samples under different conditions. (**b**) Heatmap showing the normalized expression levels of cell type marker genes in all samples used for bulk RNA-seq.


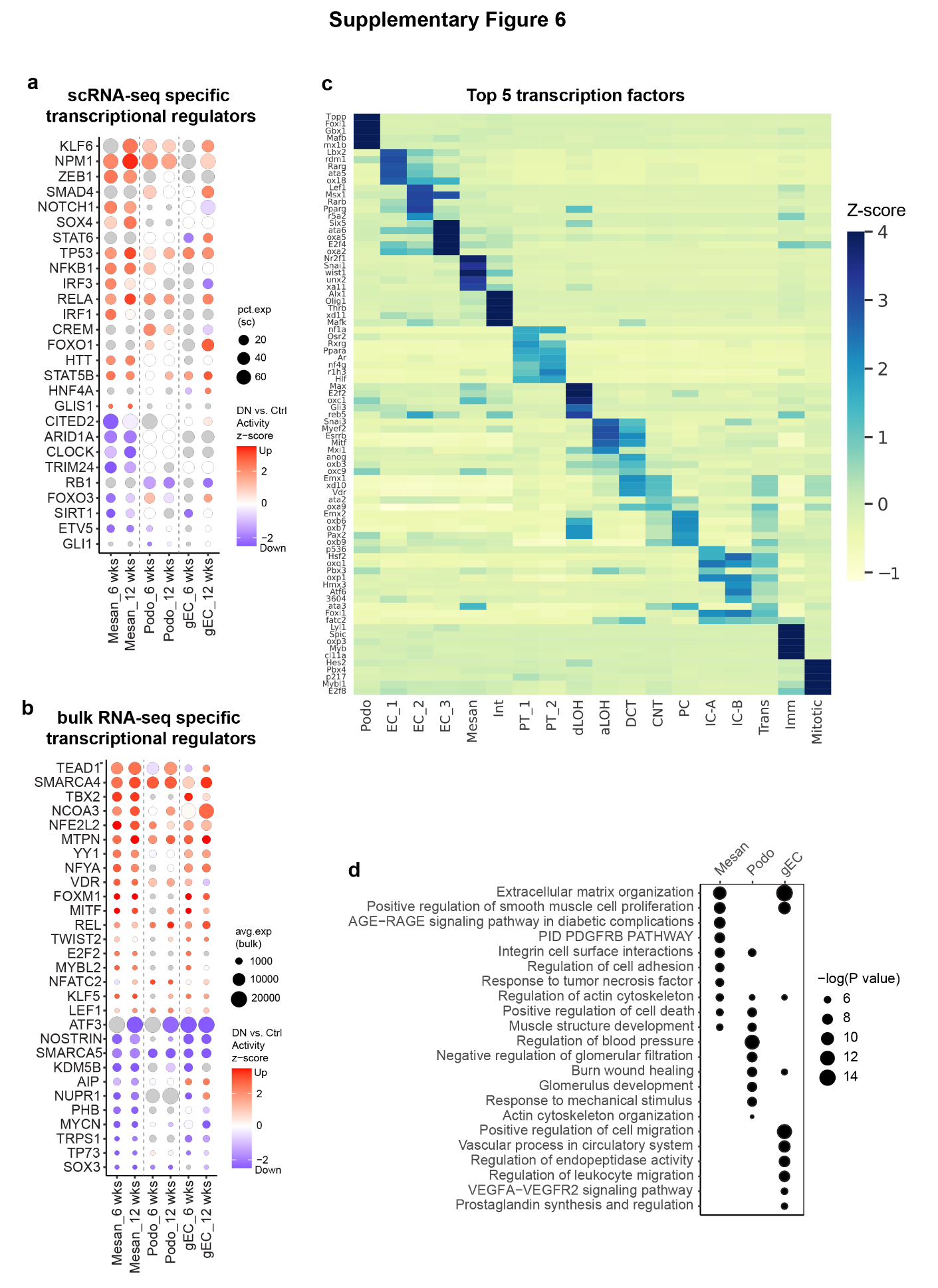


## Fig. S6: Transcriptional regulations in DN mice

(**a, b**) Dot plot displaying the changed transcriptional regulations identified in single-cell and bulk RNA-seq data estimated by IPA. Nonsignificant genes are shown in grey. (**c**) Heatmap showing the activity z scores of top 5 transcription factors estimated by SCENIC. (**d**) Top enriched pathways of MRTF transcriptional target genes in Mesan, Podo and gEC.


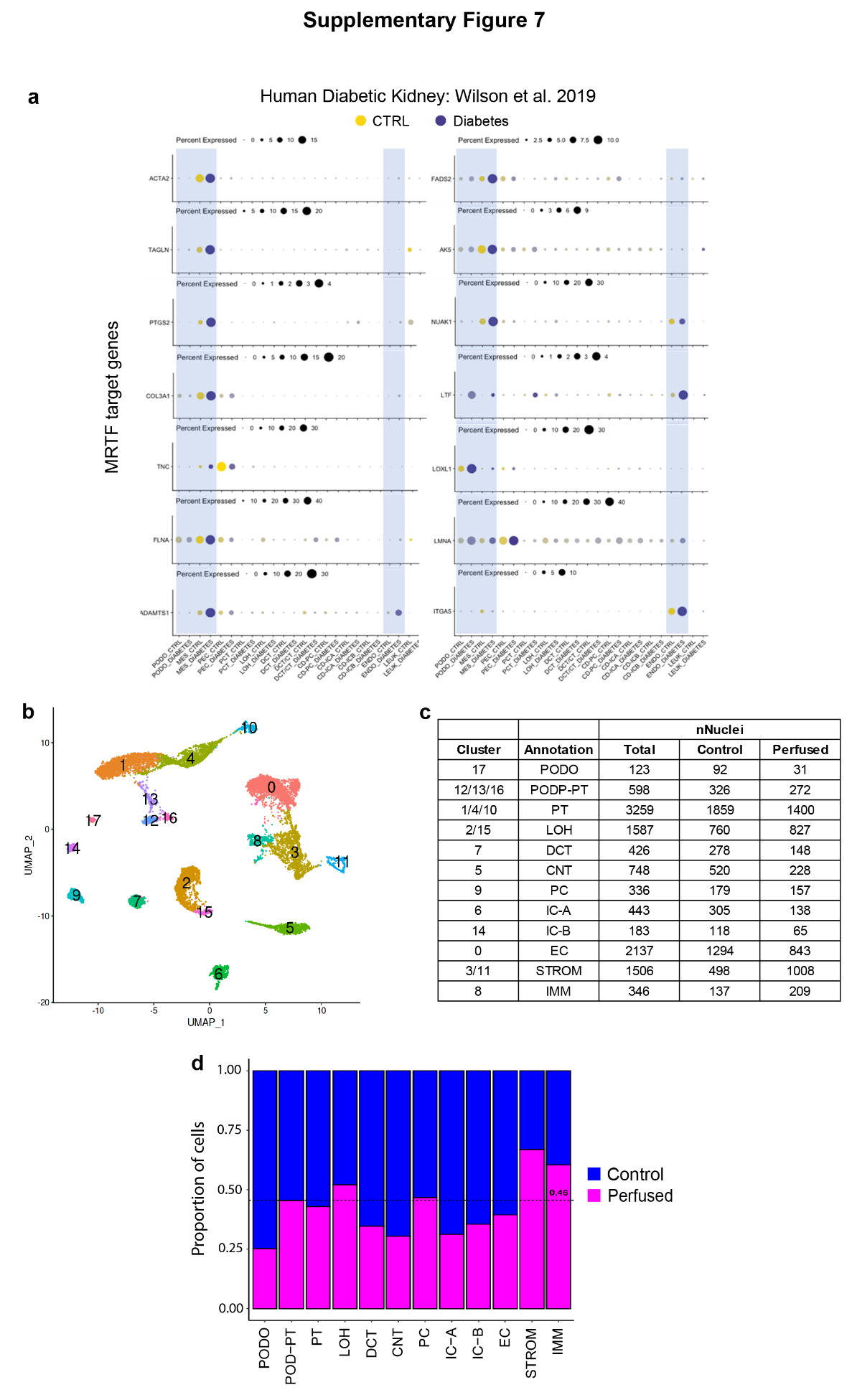


## Fig. S7: snRNA-seq of early human diabetic nephropathy and ex-vivo-perfused pig kidney tissue

(**a**) Dot plot of MRTF transcriptional target genes in control (CTRL) and early human diabetic (Diabetes) kidneys. Data from Wilson et al. (Kidney Interactive Transcriptomics: <https://humphreyslab.com/SingleCell/>) [3]. The blue background highlights control and diabetes podocytes (PODO), mesangial cells (MES) and endothelial cells (ENDO). (**b**) UMAP plot for 11,692 nuclei from perfused and control pig kidney tissue demonstrating 18 clusters (0-17). (**c**) Annotation of each cluster and the number of nuclei in each cell type separated into control and perfused groups. (**d**) Proportions of nuclei from control versus perfused pig kidney tissue per cell type. The dashed lines indicate the average proportion of all cell types.

# Supplementary methods

## FACS-sorted glomerular cells

The protocol for isolation of glomeruli from mouse kidneys and digestion of glomeruli has been described previously [4]. Briefly, glomeruli were isolated using the magnetic bead method, and the isolated glomeruli were resuspended in Collagenase II solution (300 U/ml Col II [LS004176, Worthington Biochemical], 5 U/ml Pronase E [P6911, Sigma-Aldrich], 1.25 U/ml Dispase II [D4693, Sigma-Aldrich], and 50 U/ml DNase I [Roche, 04716728001] in HBSS) and incubated for 40 min at 37 °C on a thermomixer shaker at 1,400 rpm. After digestion, single cells were sieved through a 40 µm cell strainer, and the cell suspension was centrifuged at 400 ×*g* for 4 min at 4 °C. The cells were resuspended in staining buffer (554657, BD), and the beads were removed with a magnet (DynaMag™-2 Magnet). The supernatant including all glomerular cells was taken and passed through a 35 µm cell strainer on a FACS tube. The cells were centrifuged at 400 ×*g* for 4 min at 4 °C, resuspended in staining buffer, and stained with a mix of antibodies containing PODXL- Alexa Fluor® 647(AF647) (FAB1556R, R&D), CD45-Brilliant Violet 650™(BV650) (103151, BioLegend) and CD73-PerCP/Cyanine5.5 (PerCP-Cy5.5) (127213, BioLegend) (0.2 µg per 10^6^ cells) for 30 min at 4 °C in the dark. After staining, the cells were washed with staining buffer and centrifuged at 400 ×*g* for 4 min at 4 °C. The cell pellet was resuspended in staining buffer, and DAPI (0.08 µg/ml) was added to the cells 5 min before sorting. A total of 10,000-30,000 cells were sorted in RNA lysis buffer (Qiagen) with a BD AriaFusion Sorting device. RNA was immediately isolated using an RNeasy Micro Kit (Qiagen) according to the manufacturer’s protocol.

## Urine and serum analysis

Urinary albumin was measured using a mouse albumin-specific ELISA kit (Bethyl). Urinary creatinine was measured using an enzymatic colorimetric creatinine kit (Labor+Technik, LT-CR0053) as described in the manufacturer’s instructions. Proteinuria is expressed as mg albumin/mg creatinine. Nonfasting blood glucose levels were measured using a glucometer (Accu-Chek Aviva, Roche).

## Histological and ultrastructural analysis

Kidneys were fixed in 4% paraformaldehyde, embedded in paraffin and further processed for periodic acid–Schiff staining or for immunohistochemistry using anti-Collagen IV antibody (1340-01, SouthernBiotech) and ImmPRESS Anti-Goat IgG (VEC-MP-7405-15, Vector Laboratories) according to the standard DAB staining protocol.

The specimen, fixed in formaldehyde, were put in sodium cacodylate buffer (10 min at 80°C) and 1% osmium tetroxide and sucrose (2h). After washing in cacodylate buffer and 1h contrasting in uranyl acetate, they were dehydrated in ascending ethanol series, embedded in araldite and polymerised 12 h up to 100°C. Semi thin sections were cut and stained with toluidine blue. Ultra-thin sections were cut and contrasted with lead (II) nitrate (Pb(NO3)2) and sodium citrate (C6H5Na3). Pictures were taken using a digital camera (TRS 2K-CCD) on a transmission electron microscope (Zeiss EM109).

# References

1. Adam M, Potter AS, Potter SS: **Psychrophilic proteases dramatically reduce single-cell RNA-seq artifacts: a molecular atlas of kidney development.** *Development* 2017, **144:**3625-3632.

2. van den Brink SC, Sage F, Vertesy A, Spanjaard B, Peterson-Maduro J, Baron CS, Robin C, van Oudenaarden A: **Single-cell sequencing reveals dissociation-induced gene expression in tissue subpopulations.** *Nat Methods* 2017, **14:**935-936.

3. Wilson PC, Wu H, Kirita Y, Uchimura K, Ledru N, Rennke HG, Welling PA, Waikar SS, Humphreys BD: **The single-cell transcriptomic landscape of early human diabetic nephropathy.** *Proc Natl Acad Sci U S A* 2019, **116:**19619-19625.

4. Boerries M, Grahammer F, Eiselein S, Buck M, Meyer C, Goedel M, Bechtel W, Zschiedrich S, Pfeifer D, Laloe D, et al: **Molecular fingerprinting of the podocyte reveals novel gene and protein regulatory networks.** *Kidney Int* 2013, **83:**1052-1064.
